# Soliton structures for the (3 + 1)-dimensional Painlevé integrable equation in fluid mediums

**DOI:** 10.1038/s41598-024-62314-6

**Published:** 2024-05-21

**Authors:** Jian-Guo Liu

**Affiliations:** College of Computer, Jiangxi University of Chinese Medicine, Jiangxi, 330004 China

**Keywords:** Painlevé integrable equation, Hirota bilinear form, $(G'/G)$-expansion method, Exact solutions, Applied mathematics, Computational science

## Abstract

The (3 + 1)-dimensional Painlevé integrable equation are a class of nonlinear differential equations with special properties, which play an important role in nonlinear science and are of great significance in solving various practical problems, such as many important models in fields such as quantum mechanics, statistical physics, nonlinear optics, and celestial mechanics. In this work, we utilize the Hirota bilinear form and Mathematica software to formally obtain the interaction solution among lump wave, solitary wave and periodic wave, which has not yet appeared in other literature. Additionally, using the $(G'/G)$-expansion method, we provide a rich set of exact solutions for the (3 + 1)-dimensional Painlevé integrable equation, which includes two functions with arbitrary values. This method is the first to be applied to the (3 + 1)-dimensional Painlevé integrable equation. By giving some 3D graphics and density maps, the dynamic properties are analyzed and demonstrated, which is beneficial for promoting understanding and application of the (3 + 1)-dimensional Painlevé integrable equation.

## Introduction

   In recent years, nonlinear science has developed rapidly. Many phenomena in nature can be described by nonlinear evolution equations (NLEEs). People often solve NLEEs to understand the phenomena behind the equations. For this reason, many methods for solving the exact solutions of NLEEs have been proposed, such as similarity reduction method^1^, Riemann–Hilbert approach^2^, homogeneous balance method^3^, $(G'/G)$–expansion method^4^, Riccati equation expansion method^5^, variable separation approach^6^, tanh method^7^, bilinear neural network method^8^, darboux transformation method^9^, Jacobi elliptic function method^10^, positive quadratic function method^11^ and so on. With the continuous development of these methods, researchers have obtained many exact solutions to important nonlinear evolution equations^12–15^, greatly promoting the development of nonlinear science^16–20^.

    Rogue waves are an extreme or rare event that typically occurs in fields such as oceans, optics, plasma, and finance. The characteristics of rogue waves mainly include their unusually high wave heights, and their appearance is sudden and almost irregular. Due to its extremely high wave height, it poses a serious threat to the safety of offshore buildings, ships, and personnel during navigation. For the study of rogue waves, the current focus is mainly on solving the exact solutions of rogue waves under different models^21–26^, which further reveals the essence and characteristics of rogue waves. This not only theoretically confirms the existence of rogue waves, but also lays a theoretical foundation for the generation of rogue waves in experiments. Overall, rogue waves are an extreme wave phenomenon with unique spatiotemporal distribution and statistical rules, and their research is of great significance for understanding extreme events in fields such as oceans and optics^27–31,31–34^.

    The Painlevé integrable equation also holds an important position in mathematical theory. They are closely related to mathematical fields such as special functions, elliptic functions, and orthogonal polynomials, providing new perspectives and methods for research in these fields. This paper investigates the following (3 + 1)-dimensional Painlevé integrable equation in fluid mediums^35^

1 $$\begin{aligned} &  \delta _7 u_{zz}+\delta _8 u_{yz}+\delta _5 u_{yy}+\delta _2 u_{xz}+\delta _1 u_{xy}+\delta _3 u_{xx}\nonumber \\ &  \quad +\delta _6 \left( 2 u_x^2+2 u u_{xx}\right) +\delta _4 u_{xxxx}+u_{xt}=0, \end{aligned}$$

where $u=u(x,y,z,t)$, $\delta _i(i=1,2,\ldots ,7,8)$ is arbitrary constant. Eq. (1) was first proposed by Prof. Abdul-Majid Wazwaz^35^ and played an important role in nonlinear science, such as many important models in fields such as quantum mechanics, statistical physics, nonlinear optics, and celestial mechanics. Prof. Abdul-Majid Wazwaz examined the integrability of Eq. (1) using the Painlevé analysis method and obtained the multiple soliton and lump solutions. However, the interaction solution among lump wave, solitary wave and periodic wave has not been investigated, there is no literature on the $(G'/G)$-expansion method for solving non-traveling wave exact solutions of this equation, which will be our main work.

   Substituting the logarithmic transformation $u=\frac{6 \delta _4}{\delta _6} (\ln \xi )_{xx}$ into Eq. (1), we have

2 $$\begin{aligned} &  [\delta _3 D^2_x+\delta _5 D^2_y+\delta _4 D^4_x+D_x D_t+\delta _1 D_x D_y+\delta _2 D_x D_z+D_z D_y+\frac{1}{4 \delta _5} D^2_z] \xi \cdot \xi \nonumber \\ &  \quad =\frac{\xi \xi _{zz}-\xi _z^2}{4 \delta _5}+\delta _5 \left( \xi \xi _{yy}-\xi _y^2\right) +\delta _2 \left( \xi \xi _{xz}-\xi _z \xi _x\right) +\delta _1 \left( \xi \xi _{xy}-\xi _y \xi _x\right) \nonumber \\ &  \quad \quad +\,\delta _3 \left( \xi \xi _{xx}-\xi _x^2\right) +\delta _4 \left( 3 \xi _{xx}^2-4 \xi _x \xi _{xxx}+\xi \xi _{xxxx}\right) -\xi _z \xi _y+\xi \xi _{yz}\nonumber \\ &  \quad \quad -\,\xi _t \xi _x+\xi \xi _{xt}=0, \delta _8=1, \delta _7=\frac{1}{4 \delta _5}, \end{aligned}$$

where $$\xi =\xi (x,y,z,t)$$.

   The paper is designed as follows: “Lump-soliton solution” Section obtains the interaction solution between lump wave and solitary waves based on the Hirota bilinear form, which have not been seen in other literature; “Lump-periodic solution” Section derives the interaction solution between lump wave and periodic wave via the Hirota bilinear form; [Sec Sec4] Section investigates some other types of exact solutions by utilizing the $(G'/G)$-expansion method. The obtained exact solution contains two arbitrary functions. When these two arbitrary functions take different values, we can obtain infinite exact solutions to equation (1). Compared to the previous $(G'/G)$-expansion method, we employ a unique nonlinear transformation to obtain more types of exact solutions to Eq. (1). “Conclusion” Section gives a summary.

## Lump-soliton solution

   Prof. Abdul-Majid Wazwaz discussed the lump wave solution of Eq. (1) using positive quadratic functions, but he did not discuss the interaction between lump wave and other solitons. By studying the interaction solutions between lump wave and soliton, new wave phenomena and patterns may be discovered, which may help promote scientific research and technological applications in related fields, making it very meaningful. To this end, we make a supplement on the basis of Ref.^35^, assuming that Eq. (2) has the following interaction solutions

3 $$\begin{aligned} &  \xi =\eta _5+k_1 e^{\eta _4 t+\eta _1 x+\eta _2 y+\eta _3 z}+k_2 e^{-\eta _4 t-\eta _1 x-\eta _2 y-\eta _3 z}\nonumber \\ &  \quad +\left( \mathcal {G}_5+\mathcal {G}_4 t+\mathcal {G}_1 x+\mathcal {G}_2 y+\mathcal {G}_3 z\right) ^2+\left( t \tau _4+\tau _5+\tau _1 x+\tau _2 y+\tau _3 z\right) ^2, \end{aligned}$$

where $\mathcal {G}_i$, $\tau _i$, $\eta _i(i=1,2,3,4,5)$ and $k_j(j=1,2)$ are unknown constants. By substituting Eq. (3) into Eq. (2), we get the following algebraic solution

4 $$\begin{aligned} &  \eta _5=\frac{\left( \mathcal {G}_1^2+\tau _1^2\right) ^2+\eta _1^4 k_1 k_2}{\eta _1^2 \left( \mathcal {G}_1^2+\tau _1^2\right) }, \tau _4=\tau _1 \left( -3 \delta _4 \eta _1^2-\delta _3\right) , \tau _2=-\frac{\mathcal {G}_1 \mathcal {G}_2}{\tau _1},\nonumber \\ &  \quad \mathcal {G}_3=-\frac{\tau _1 \tau _3}{\mathcal {G}_1}, \mathcal {G}_4=-\mathcal {G}_1 \left( 3 \delta _4 \eta _1^2+\delta _3\right) , \tau _3=\frac{\delta _1 \mathcal {G}_1 \mathcal {G}_2}{\delta _2 \tau _1}, \eta _3=-2 \delta _5 \eta _2,\nonumber \\ &  \quad \eta _4=-\eta _1 \left( \delta _4 \eta _1^2+\delta _3\right) -\left( \delta _1-2 \delta _2 \delta _5\right) \eta _2, \mathcal {G}_2=\frac{2 \sqrt{3} \delta _2 \sqrt{-\delta _4 \delta _5} \eta _1 \tau _1}{\delta _1-2 \delta _2 \delta _5}. \end{aligned}$$

**Figure 1:**
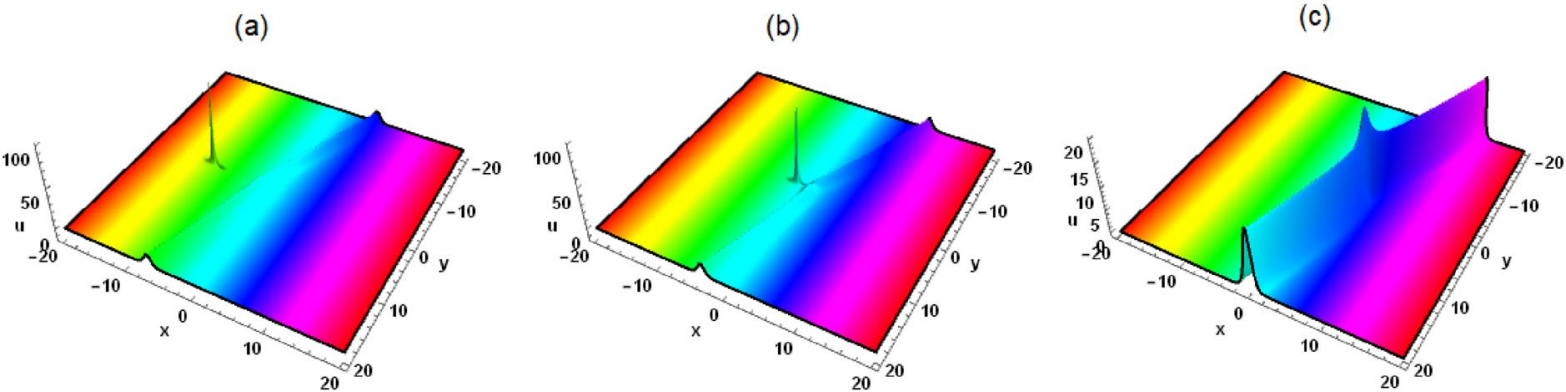
Solution (5) with $$\mathcal {G}_1=\tau _1=\tau _5=\eta _2=k_1=\delta _1=\delta _2 =\delta _3=\delta _4=\delta _6=1$$, $$\mathcal {G}_5=2$$, $$\eta _1=3$$, $$\delta _5=-1$$, $$k_2=z=0$$, (**a**) $$t=-0.3$$, (**b**) $$t=0$$, (**c**) $$t=0.3$$.

**Figure 2:**
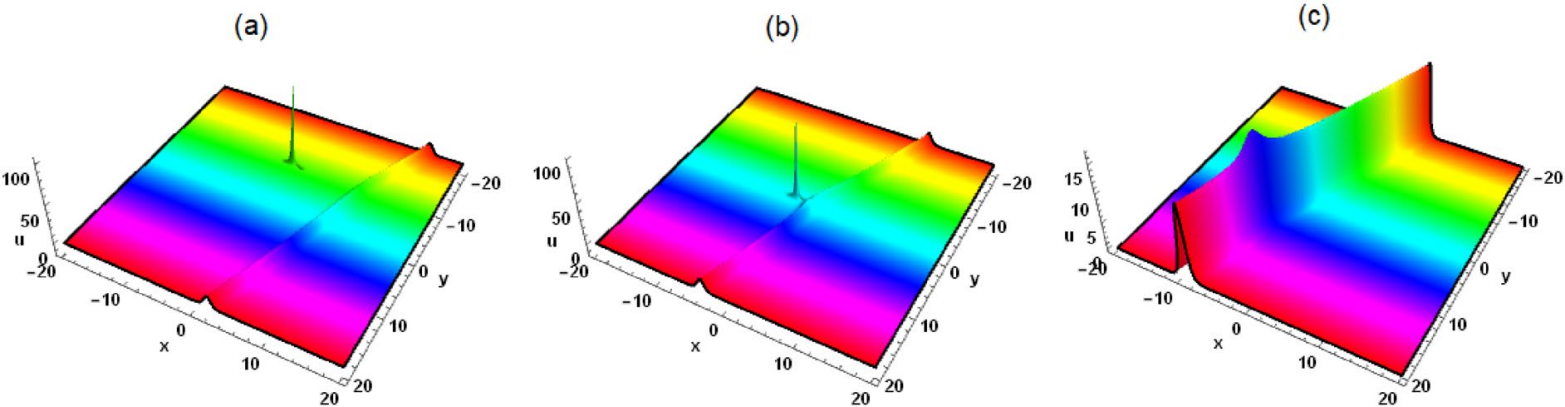
Solution (5) with $$\mathcal {G}_1=\tau _1=\tau _5=\eta _2=k_1=\delta _1=\delta _2 =\delta _3=\delta _4=\delta _6=1$$, $$\mathcal {G}_5=2$$, $$\eta _1=3$$, $$\delta _5=-1$$, $$k_2=t=0$$, (**a**) $$z=-8$$, (**b**) $$z=0$$, (**c**) $$z=8$$.

Substituting Eqs. (3) and (4) into the logarithmic transformation $u=\frac{6 \delta _4}{\delta _6} (\ln \xi )_{xx}$, the interaction solution between lump wave and solitons is presented as follows

5 $$\begin{aligned} &  u=[6 \delta _4 [[2 \mathcal {G}_1^2+e^{x \eta _1+y \eta _2-2 z \delta _5 \eta _2+t [-\eta _1 \left( \delta _4 \eta _1^2+\delta _3\right) -\left( \delta _1-2 \delta _2 \delta _5\right) \eta _2]} k_1 \eta _1^2\nonumber \\ &  \quad \quad +\,e^{-x \eta _1-y \eta _2+2 z \delta _5 \eta _2-t [-\eta _1 \left( \delta _4 \eta _1^2+\delta _3\right) -\left( \delta _1-2 \delta _2 \delta _5\right) \eta _2]} k_2 \eta _1^2+2 \tau _1^2]/[[x \mathcal {G}_1-t (3 \delta _4 \eta _1^2\nonumber \\ &  \quad \quad +\,\delta _3) \mathcal {G}_1+\mathcal {G}_5-\frac{2 \sqrt{3} z \delta _1 \sqrt{-\delta _4 \delta _5} \eta _1 \tau _1}{\delta _1-2 \delta _2 \delta _5}+\frac{2 \sqrt{3} y \delta _2 \sqrt{-\delta _4 \delta _5} \eta _1 \tau _1}{\delta _1-2 \delta _2 \delta _5}]^2\nonumber \\ &  \quad \quad +\,\frac{[2 \sqrt{3} \mathcal {G}_1 \left( z \delta _1-y \delta _2\right) \sqrt{-\delta _4 \delta _5} \eta _1+\left( \delta _1-2 \delta _2 \delta _5\right) [[x-t \left( 3 \delta _4 \eta _1^2+\delta _3\right) ] \tau _1+\tau _5]]^2}{\left( \delta _1-2 \delta _2 \delta _5\right) ^2}\nonumber \\ &  \quad \quad +\,e^{x \eta _1+y \eta _2-2 z \delta _5 \eta _2+t [-\eta _1 \left( \delta _4 \eta _1^2+\delta _3\right) -\left( \delta _1-2 \delta _2 \delta _5\right) \eta _2]} k_1\nonumber \\ &  \quad \quad +\,e^{-x \eta _1-y \eta _2+2 z \delta _5 \eta _2-t [-\eta _1 \left( \delta _4 \eta _1^2+\delta _3\right) -\left( \delta _1-2 \delta _2 \delta _5\right) \eta _2]} k_2+\frac{k_1 k_2 \eta _1^4+\left( \mathcal {G}_1^2+\tau _1^2\right) ^2}{\eta _1^2 \left( \mathcal {G}_1^2+\tau _1^2\right) }]\nonumber \\ &  \quad \quad -\,[[e^{x \eta _1+y \eta _2-2 z \delta _5 \eta _2+t \left( -\eta _1 \left( \delta _4 \eta _1^2+\delta _3\right) -\left( \delta _1-2 \delta _2 \delta _5\right) \eta _2\right) } k_1 \eta _1\nonumber \\ &  \quad \quad -\,e^{-x \eta _1-y \eta _2+2 z \delta _5 \eta _2-t \left( -\eta _1 \left( \delta _4 \eta _1^2+\delta _3\right) -\left( \delta _1-2 \delta _2 \delta _5\right) \eta _2\right) } k_2 \eta _1+2 \mathcal {G}_1 [x \mathcal {G}_1-t (3 \delta _4 \eta _1^2\nonumber \\ &  \quad \quad +\,\delta _3) \mathcal {G}_1+\mathcal {G}_5-\frac{2 \sqrt{3} z \delta _1 \sqrt{-\delta _4 \delta _5} \eta _1 \tau _1}{\delta _1-2 \delta _2 \delta _5}+\frac{2 \sqrt{3} y \delta _2 \sqrt{-\delta _4 \delta _5} \eta _1 \tau _1}{\delta _1-2 \delta _2 \delta _5}]\nonumber \\ &  \quad \quad +\,[2 \tau _1 [2 \sqrt{3} \mathcal {G}_1 \left( z \delta _1-y \delta _2\right) \sqrt{-\delta _4 \delta _5} \eta _1+\left( \delta _1-2 \delta _2 \delta _5\right) [[x-t \left( 3 \delta _4 \eta _1^2+\delta _3\right) ] \tau _1\nonumber \\ &  \quad \quad +\,\tau _5]]]/[\delta _1-2 \delta _2 \delta _5]]^2]/[[[x \mathcal {G}_1-t \left( 3 \delta _4 \eta _1^2+\delta _3\right) \mathcal {G}_1+\mathcal {G}_5-\frac{2 \sqrt{3} z \delta _1 \sqrt{-\delta _4 \delta _5} \eta _1 \tau _1}{\delta _1-2 \delta _2 \delta _5}\nonumber \\ &  \quad \quad +\,\frac{2 \sqrt{3} y \delta _2 \sqrt{-\delta _4 \delta _5} \eta _1 \tau _1}{\delta _1-2 \delta _2 \delta _5}]^2+[[2 \sqrt{3} \mathcal {G}_1 \left( z \delta _1-y \delta _2\right) \sqrt{-\delta _4 \delta _5} \eta _1+\left( \delta _1-2 \delta _2 \delta _5\right) [[x\nonumber \\ &  \quad \quad -\,t \left( 3 \delta _4 \eta _1^2+\delta _3\right) ] \tau _1+\tau _5]]^2]/\left( \delta _1-2 \delta _2 \delta _5\right) ^2\nonumber \\ &  \quad \quad +\,e^{x \eta _1+y \eta _2-2 z \delta _5 \eta _2+t [-\eta _1 \left( \delta _4 \eta _1^2+\delta _3\right) -\left( \delta _1-2 \delta _2 \delta _5\right) \eta _2]} k_1+\frac{k_1 k_2 \eta _1^4+\left( \mathcal {G}_1^2+\tau _1^2\right) ^2}{\eta _1^2 \left( \mathcal {G}_1^2+\tau _1^2\right) }\nonumber \\ &  \quad \quad +\,e^{-x \eta _1-y \eta _2+2 z \delta _5 \eta _2-t [-\eta _1 \left( \delta _4 \eta _1^2+\delta _3\right) -\left( \delta _1-2 \delta _2 \delta _5\right) \eta _2]} k_2]^2]]]/\delta _6. \end{aligned}$$

**Figure 3:**
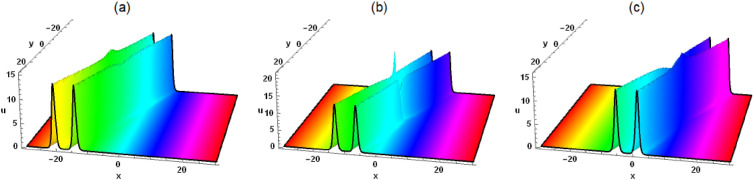
Solution (5) with $$\mathcal {G}_1=\tau _1=\tau _5=\eta _2=k_1=\delta _1=\delta _2 =\delta _3=\delta _4=\delta _6=1$$, $$\mathcal {G}_5=k_2=2$$, $$\eta _1=3$$, $$\delta _5=-1$$, $$z=0$$, (**a**) $$t=-0.7$$, (**b**) $$t=0$$, (**c**) $$t=0.7$$.

**Figure 4:**
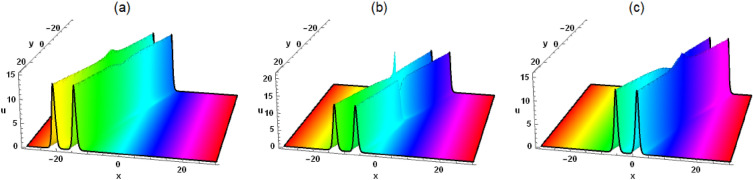
Figure 3 corresponding density map.

When $k_1=k_2=0$, Eq. (5) represents the lump wave, which has been discussed in detail in Ref.^5^. When $k_2=0$ and $k_1\ne 0$, Eq. (5) depicts the interaction between lump wave and one solitary wave. As can be seen from Fig. 1, the solitary wave propagates in the front and the lump wave propagates in the back along the $x-axis$ in the same direction in the forward direction, and the speed and amplitude of the lump wave are both greater than that of the solitary wave. After a period of time, the lump wave catches up with the solitary wave and spreads forward after joining together. The amplitude of the lump wave decreases obviously, which belongs to inelastic collision. However, when *z* is taken to different values, we can observe that the solitary wave and the lump wave begin to propagate towards each other, and then join together and continue to propagate forward, and the amplitude of the lump wave also decreases significantly. When $k_2$ and $k_1$ are not equal to zero at the same time, Eq. (5) describes the interaction between lump wave and two solitary waves (see Figs. 3 and 4). As can be seen from Figs. 3 and 4, the lump wave suddenly appears on one of the solitons, and then slowly moves to the other soliton. During the moving process, the amplitude gradually increases until it reaches the maximum value at some point, and then transfers to the other soliton and propagates forward together, and the amplitude becomes smaller.

### Ethical standard

The authors state that this research complies with ethical standards. This research does not involve either human participants or animals.

## Lump-periodic solution

    The interaction solution between lump wave and periodic wave is of great significance in multiple fields, especially in oceanography, water wave dynamics, and physics. To study the interaction solution of the Painlevé integrable equation, we assume that Eq. (2) has the following interaction solution between lump wave and periodic wave

6 $$\begin{aligned} &  \xi =\eta _9+k_2 \sin \left( \eta _8 t+\eta _5 x+\eta _6 y+\eta _7 z\right) +k_1 \cos \left( \eta _4 t+\eta _1 x+\eta _2 y+\eta _3 z\right) \nonumber \\ &  \quad \quad +\left( \mathcal {G}_5+\mathcal {G}_4 t+\mathcal {G}_1 x+\mathcal {G}_2 y+\mathcal {G}_3 z\right) ^2+\left( t \tau _4+\tau _5+\tau _1 x+\tau _2 y+\tau _3 z\right) ^2, \end{aligned}$$

where $\eta _i(i=1,2,\ldots ,8,9)$ is unknown constant. By substituting Eq. (6) into Eq. (2), we have

7 $$\begin{aligned} &  \eta _9=-\frac{4 \left( \mathcal {G}_1^2+\tau _1^2\right) ^2+\eta _1^4 \left( k_1^2+k_2^2\right) }{4 \eta _1^2 \left( \mathcal {G}_1^2+\tau _1^2\right) }, \tau _4=\tau _1 \left( -3 \delta _4 \eta _1^2-\delta _3\right) , \nonumber \\ &  \quad \mathcal {G}_3=-\frac{\tau _1 \tau _3}{\mathcal {G}_1}, \mathcal {G}_4=-\mathcal {G}_1 \left( \delta _3-3 \delta _4 \eta _1^2\right) , \tau _3=\frac{\delta _1 \mathcal {G}_1 \mathcal {G}_2}{\delta _2 \tau _1}, \eta _3=\eta _7=-2 \delta _5 \eta _6,\nonumber \\ &  \quad \eta _2=\eta _6, \eta _5=\eta _1, \mathcal {G}_2=\frac{2 \sqrt{3} \delta _2 \sqrt{\delta _4 \delta _5} \eta _1 \tau _1}{\delta _1-2 \delta _2 \delta _5}, \tau _2=-\frac{\mathcal {G}_1 \mathcal {G}_2}{\tau _1},\nonumber \\ &  \quad \eta _4=\eta _8=\delta _4 \eta _1^3-\delta _3 \eta _1-\left( \delta _1-2 \delta _2 \delta _5\right) \eta _6. \end{aligned}$$

**Figure 5:**
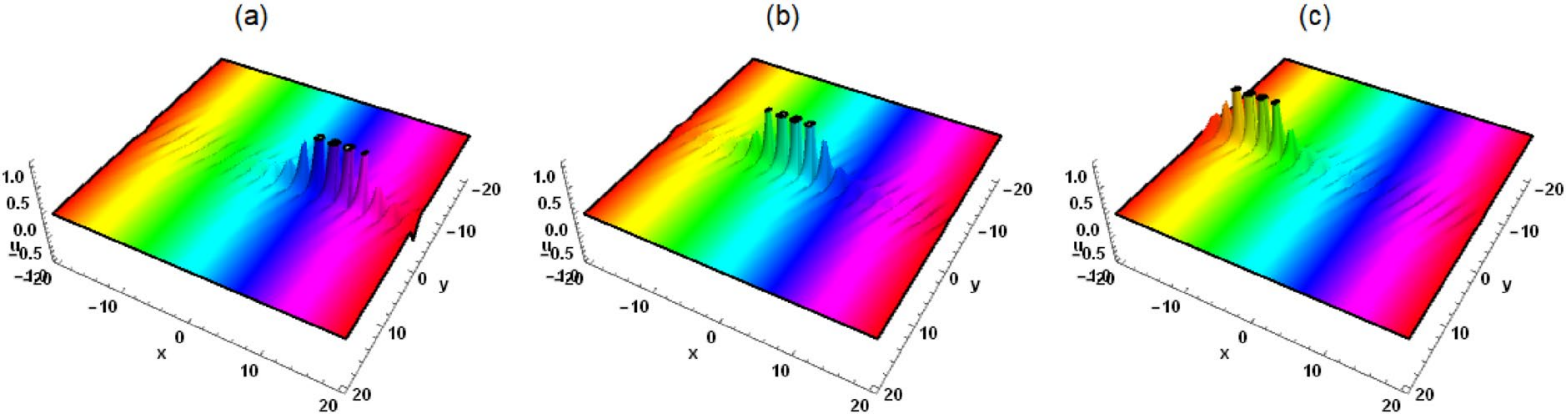
Solution (8) with $$\mathcal {G}_1=\tau _1=\tau _5=k_1=\delta _1=\delta _2 =\delta _3=\delta _4=\delta _5=1$$, $$\mathcal {G}_5=2$$, $$\eta _1=3$$, $$\eta _6=\delta _6=1$$, $$k_2=z=0$$, (**a**) $$t=-0.4$$, (**b**) $$t=0$$, (**c**) $$t=0.4$$.

**Figure 6:**
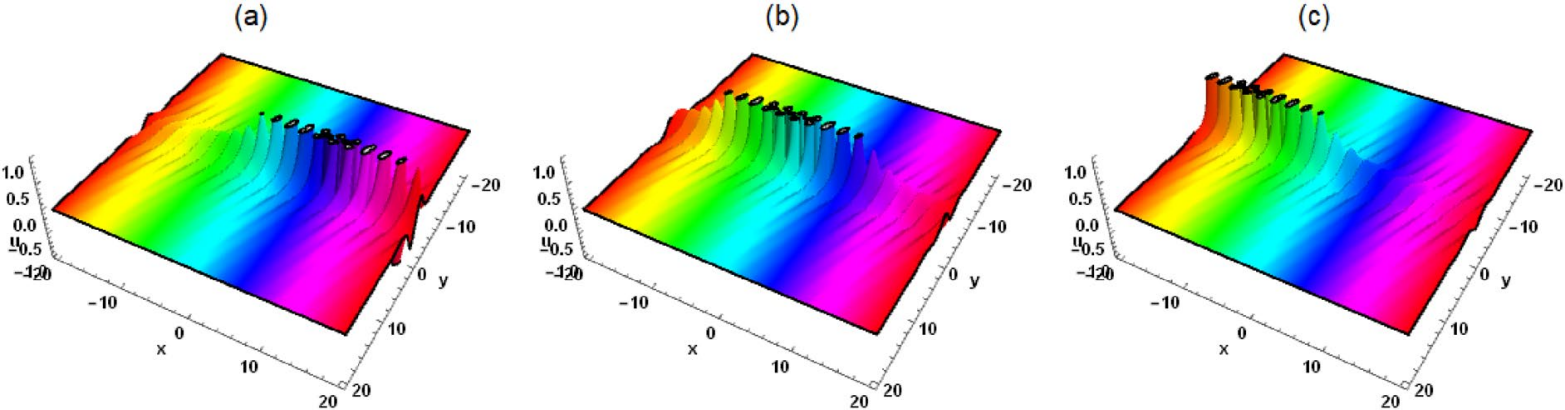
Solution (8) with $$\mathcal {G}_1=\tau _1=\tau _5=k_1=\delta _1=\delta _2 =\delta _3=\delta _4=\delta _5=1$$, $$\mathcal {G}_5=2$$, $$\eta _1=3$$, $$\eta _6=\delta _6=1$$, $$k_2=4$$, $$z=0$$, (**a**) $$t=-0.4$$, (**b**) $$t=0$$, (**c**) $$t=0.4$$.

Substituting Eqs. (6) and (7) into the logarithmic transformation $u=\frac{6 \delta _4}{\delta _6} (\ln \xi )_{xx}$, the interaction solution between lump wave and periodic wave is given as follows

8 $$\begin{aligned} &  u=[6 \delta _4 [[2 \mathcal {G}_1^2-\cos [x \eta _1+y \eta _6-2 z \delta _5 \eta _6+t [\delta _4 \eta _1^3-\delta _3 \eta _1-\left( \delta _1-2 \delta _2 \delta _5\right) \eta _6]]\nonumber \\ &  \quad \quad k_1 \eta _1^2-\sin [x \eta _1+y \eta _6-2 z \delta _5 \eta _6+t [\delta _4 \eta _1^3-\delta _3 \eta _1-\left( \delta _1-2 \delta _2 \delta _5\right) \eta _6]] k_2 \eta _1^2\nonumber \\ &  \quad \quad +\,2 \tau _1^2]/[[x \mathcal {G}_1-t \left( \delta _3-3 \delta _4 \eta _1^2\right) \mathcal {G}_1+\mathcal {G}_5-\frac{2 \sqrt{3} z \delta _1 \sqrt{\delta _4 \delta _5} \eta _1 \tau _1}{\delta _1-2 \delta _2 \delta _5}\nonumber \\ &  \quad \quad +\,\frac{2 \sqrt{3} y \delta _2 \sqrt{\delta _4 \delta _5} \eta _1 \tau _1}{\delta _1-2 \delta _2 \delta _5}]^2+[[2 \sqrt{3} \mathcal {G}_1 \left( z \delta _1-y \delta _2\right) \sqrt{\delta _4 \delta _5} \eta _1+\left( \delta _1-2 \delta _2 \delta _5\right) \nonumber \\ &  \quad \quad [\left( 3 t \delta _4 \eta _1^2+x-t \delta _3\right) \tau _1+\tau _5]]^2]/\left( \delta _1-2 \delta _2 \delta _5\right) ^2+\cos [x \eta _1+y \eta _6-2 z \delta _5 \eta _6\nonumber \\ &  \quad \quad +\,t [\delta _4 \eta _1^3-\delta _3 \eta _1-\left( \delta _1-2 \delta _2 \delta _5\right) \eta _6]] k_1+\sin [x \eta _1+y \eta _6-2 z \delta _5 \eta _6\nonumber \\ &  \quad \quad +\,t [\delta _4 \eta _1^3-\delta _3 \eta _1-\left( \delta _1-2 \delta _2 \delta _5\right) \eta _6]] k_2-\frac{\left( k_1^2+k_2^2\right) \eta _1^4+4 \left( \mathcal {G}_1^2+\tau _1^2\right) ^2}{4 \eta _1^2 \left( \mathcal {G}_1^2+\tau _1^2\right) }]\nonumber \\ &  \quad \quad -\,[[-\sin [x \eta _1+y \eta _6-2 z \delta _5 \eta _6+t \left( \delta _4 \eta _1^3-\delta _3 \eta _1-\left( \delta _1-2 \delta _2 \delta _5\right) \eta _6\right) ] k_1 \eta _1\nonumber \\ &  \quad \quad +\,\cos [x \eta _1+y \eta _6-2 z \delta _5 \eta _6+t \left( \delta _4 \eta _1^3-\delta _3 \eta _1-\left( \delta _1-2 \delta _2 \delta _5\right) \eta _6\right) ] k_2 \eta _1\nonumber \\ &  \quad \quad +\,2 \mathcal {G}_1 [x \mathcal {G}_1-t \left( \delta _3-3 \delta _4 \eta _1^2\right) \mathcal {G}_1+\mathcal {G}_5-\frac{2 \sqrt{3} z \delta _1 \sqrt{\delta _4 \delta _5} \eta _1 \tau _1}{\delta _1-2 \delta _2 \delta _5}\nonumber \\ &  \quad \quad +\,\frac{2 \sqrt{3} y \delta _2 \sqrt{\delta _4 \delta _5} \eta _1 \tau _1}{\delta _1-2 \delta _2 \delta _5}]+[2 \tau _1 [2 \sqrt{3} \mathcal {G}_1 \left( z \delta _1-y \delta _2\right) \sqrt{\delta _4 \delta _5} \eta _1+\left( \delta _1-2 \delta _2 \delta _5\right) \nonumber \\ &  \quad \quad [\left( 3 t \delta _4 \eta _1^2+x-t \delta _3\right) \tau _1+\tau _5]]]/[\delta _1-2 \delta _2 \delta _5]]^2]/[[[x \mathcal {G}_1-t \left( \delta _3-3 \delta _4 \eta _1^2\right) \mathcal {G}_1\nonumber \\ &  \quad \quad +\,\mathcal {G}_5-\frac{2 \sqrt{3} z \delta _1 \sqrt{\delta _4 \delta _5} \eta _1 \tau _1}{\delta _1-2 \delta _2 \delta _5}+\frac{2 \sqrt{3} y \delta _2 \sqrt{\delta _4 \delta _5} \eta _1 \tau _1}{\delta _1-2 \delta _2 \delta _5}]^2+[[2 \sqrt{3} \mathcal {G}_1 \left( z \delta _1-y \delta _2\right) \nonumber \\ &  \quad \quad \sqrt{\delta _4 \delta _5} \eta _1+\left( \delta _1-2 \delta _2 \delta _5\right) [\left( 3 t \delta _4 \eta _1^2+x-t \delta _3\right) \tau _1+\tau _5]]^2]/\left( \delta _1-2 \delta _2 \delta _5\right) ^2\nonumber \\ &  \quad \quad +\,\cos [x \eta _1+y \eta _6-2 z \delta _5 \eta _6+t [\delta _4 \eta _1^3-\delta _3 \eta _1-\left( \delta _1-2 \delta _2 \delta _5\right) \eta _6]] k_1\nonumber \\ &  \quad \quad +\,\sin [x \eta _1+y \eta _6-2 z \delta _5 \eta _6+t [\delta _4 \eta _1^3-\delta _3 \eta _1-\left( \delta _1-2 \delta _2 \delta _5\right) \eta _6]] k_2\nonumber \\ &  \quad \quad -\,\frac{\left( k_1^2+k_2^2\right) \eta _1^4+4 \left( \mathcal {G}_1^2+\tau _1^2\right) ^2}{4 \eta _1^2 \left( \mathcal {G}_1^2+\tau _1^2\right) }]^2]]]/\delta _6. \end{aligned}$$

As can be seen from Figs. 5 and 6, the lump and periodic waves are always entangled and propagate forward. Fig. 5 illustrates the interaction between a lump wave and a cosine periodic wave in the $x-y$ plane. During propagation, the amplitude of the lump wave continuously changes, indicating that it is an inelastic collision and energy is transferred. Figure 6 shows the interaction among lump wave, sine and cosine periodic wave. The amplitudes of the two different periodic waves have changed, indicating an inelastic collision.

##  The $(G'/G)$-expansion method

   Based on the $(G'/G)$-expansion method, we directly assume that Eq. (1) has the exact solution of the following form

9 $$\begin{aligned} &  u=\sum _{i=0}^2 a_i\,(\frac{G'}{G})^i,\nonumber \\ &  \xi =f(c z+y)+h(t)+x \phi \end{aligned}$$

where $G = G(\xi )$, $\phi$ and *c* are unknown constants, $a_i(i=0,1,2)=a_i(x,y,z,t)$, $f(c z+y)$ and *h*(*t*) are functions to be determined later. The *G* satisfies

10 $$\begin{aligned} G''+\lambda \,G'+\mu \,G=0, \end{aligned}$$

where $\lambda$ and $\mu$ are arbitrary constants. Eq. (10) has the following solutions

*Case 1:* When $\lambda ^2-4 \mu >0$, $\frac{G'}{G}$ satisfies

11 $$\begin{aligned} \frac{G'}{G}=-\frac{\lambda }{2}+\tau _1 \frac{C_1 \cosh (\tau _1 \xi )+C_2 \sinh (\tau _1 \xi )}{C_1 \sinh (\tau _1 \xi )+C_2 \cosh (\tau _1 \xi )}, \end{aligned}$$

where $\tau _1=\frac{\sqrt{\lambda ^2-4 \mu } }{2}$ and $C_1$, and $C_2$ are arbitrary constants.

*Case 2:* When $\lambda ^2-4 \mu <0$, we have

12 $$\begin{aligned} \frac{G'}{G}=-\frac{\lambda }{2}+\tau _2 \frac{-C_1 \sin (\tau _2 \xi )+C_2 \cos (\tau _2 \xi )}{C_1 \cos (\tau _2 \xi )+C_2 \sin (\tau _2 \xi )}, \end{aligned}$$

where $$\tau _2=\frac{\sqrt{-\lambda ^2+4 \mu } }{2}$$.

*Case 3:* When $\lambda ^2-4 \mu =0$, we get

13 $$\begin{aligned} \frac{G'}{G}=-\frac{\lambda }{2}+\frac{C_2}{C_1+C_2 \xi }. \end{aligned}$$

Compared to the linear transformation commonly used in the $(G'/G)$-expansion method, we have added two nonlinear functions $f(c z+y)$ and *h*(*t*) to form a nonlinear transformation, which can better handle the coefficients in Eq. (1) and obtain more exact solutions for different types of traveling and non-traveling waves. This is something that the original $(G'/G)$-expansion method cannot achieve. There is no unified form for new nonlinear transformations, and different nonlinear evolution equations require the construction of different nonlinear transformations, which is quite inconvenient. Substituting Eqs. (9) and (10) into Eq. (1), we derive *Case 1:*

14 $$\begin{aligned} &  a_2=-\frac{6 \delta _4 \phi ^2}{\delta _6}, a_1=-\frac{6 \delta _4 \lambda \phi ^2}{\delta _6}, c=\frac{\sqrt{\delta _8^2-4 \delta _5 \delta _7}-\delta _8}{2 \delta _7},\nonumber \\ &  \quad a_0=-[f'(c z+y) [\phi \left( c \delta _2+\delta _1\right) +[c \left( c \delta _7+\delta _8\right) +\delta _5] f'(c z+y)]\nonumber \\ &  \quad \quad \quad +\,\delta _4 \phi ^4 \left( \lambda ^2+8 \mu \right) +\delta _3 \phi ^2+\phi h'(t)]/(2 \delta _6 \phi ^2). \end{aligned}$$

Substituting Eqs. (13) and (14) into Eq. (9), the rational solution of Eq. (1) is obtained as follows

15 $$\begin{aligned} &  u=-[4 \delta _4 \phi ^3 [\frac{6 C_2^2}{[C_2 [f\left( y+\frac{\left( \sqrt{\delta _8^2-4 \delta _5 \delta _7}-\delta _8\right) z}{2 \delta _7}\right) +h(t)+x \phi ]+C_1]^2}-\lambda ^2+4 \mu ]\nonumber \\ &  \quad \quad +\,2 \delta _3 \phi +[2 \delta _1 \delta _7 f'\left( y+\frac{\left( \sqrt{\delta _8^2-4 \delta _5 \delta _7}-\delta _8\right) z}{2 \delta _7}\right) +\delta _2 (\sqrt{\delta _8^2-4 \delta _5 \delta _7}-\delta _8)\nonumber \\ &  \quad \quad f'\left( y+\frac{\left( \sqrt{\delta _8^2-4 \delta _5 \delta _7}-\delta _8\right) z}{2 \delta _7}\right) +2 \delta _7 h'(t)]/\delta _7]/(4 \delta _6 \phi ). \end{aligned}$$

Substituting Eqs. (11) and (14) into Eq. (9), the hyperbolic function solution of Eq. (1) is given as follows

16 $$\begin{aligned} &  u=[2 \delta _4 \phi ^3 \left( \lambda ^2-4 \mu \right) [-[3 \left( C_1^2-C_2^2\right) ]/[[C_1 \sinh [\frac{1}{2} \sqrt{\lambda ^2-4 \mu } [f(y\nonumber \\ &  \quad \quad +\,\frac{\left( \sqrt{\delta _8^2-4 \delta _5 \delta _7}-\delta _8\right) z}{2 \delta _7})+h(t)+x \phi ]]+C_2 \cosh [\frac{1}{2} \sqrt{\lambda ^2-4 \mu }\nonumber \\ &  \quad \quad [f\left( y+\frac{\left( \sqrt{\delta _8^2-4 \delta _5 \delta _7}-\delta _8\right) z}{2 \delta _7}\right) +h(t)+x \phi ]]]^2]-1]-2 \delta _3 \phi \nonumber \\ &  \quad \quad +\,[-2 \delta _1 \delta _7 f'\left( y+\frac{\left( \sqrt{\delta _8^2-4 \delta _5 \delta _7}-\delta _8\right) z}{2 \delta _7}\right) +\delta _2 \left( \delta _8-\sqrt{\delta _8^2-4 \delta _5 \delta _7}\right) \nonumber \\ &  \quad \quad f'\left( y+\frac{\left( \sqrt{\delta _8^2-4 \delta _5 \delta _7}-\delta _8\right) z}{2 \delta _7}\right) -2 \delta _7 h'(t)]/\delta _7]/(4 \delta _6 \phi ). \end{aligned}$$

**Figure 7:**
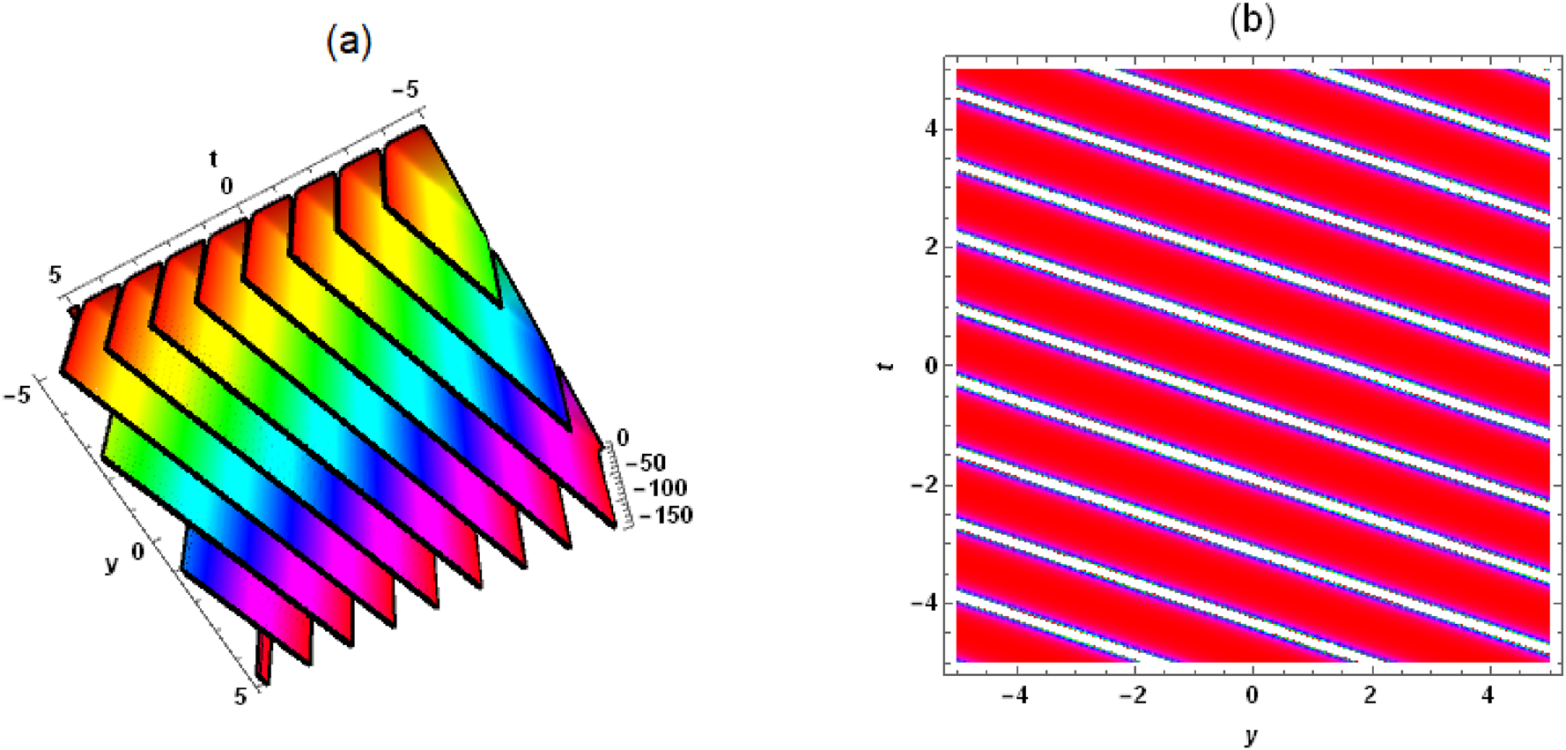
Solution (17) with $\delta _1=\delta _2 =\delta _3=\delta _4=\delta _5=1$, $C_2=-1$, $\delta _8=2$, $C_1=\delta _6=\delta _7=\lambda =\mu =1$, $\phi =1/4$, $z=x=0$, (**a**) three-dimensional plot, (**b**) density plot.

**Figure 8:**
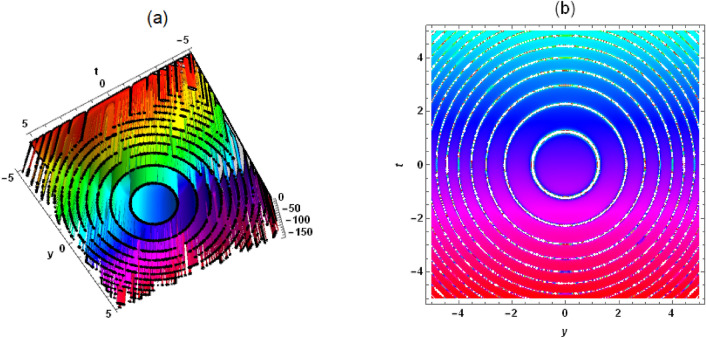
Solution (17) with $\delta _1=\delta _2 =\delta _3=\delta _4=\delta _5=1$, $C_2=-1$, $\delta _8=2$, $C_1=\delta _6=\delta _7=\lambda =\mu =1$, $\phi =1/4$, $z=x=0$, (**a**) three-dimensional plot, (**b**) density.

Substituting Eqs. (12) and (14) into Eq. (9), the periodic solution of Eq. (1) is presented as follows

17 $$\begin{aligned} &  u=[2 \delta _4 \phi ^3 \left( \lambda ^2-4 \mu \right) [[3 \left( C_1^2+C_2^2\right) ]/[[C_2 \sin [\frac{1}{2} \sqrt{4 \mu -\lambda ^2}\nonumber \\ &  \quad \quad [f\left( y+\frac{\left( \sqrt{\delta _8^2-4 \delta _5 \delta _7}-\delta _8\right) z}{2 \delta _7}\right) +h(t)+x \phi ]]+C_1 \cos [\frac{1}{2} \sqrt{4 \mu -\lambda ^2} \nonumber \\ &  \quad \quad [f\left( y+\frac{\left( \sqrt{\delta _8^2-4 \delta _5 \delta _7}-\delta _8\right) z}{2 \delta _7}\right) +h(t)+x \phi ]]]^2]-1]-2 \delta _3 \phi \nonumber \\ &  \quad \quad +\,[-2 \delta _1 \delta _7 f'\left( y+\frac{\left( \sqrt{\delta _8^2-4 \delta _5 \delta _7}-\delta _8\right) z}{2 \delta _7}\right) +\delta _2 \left( \delta _8-\sqrt{\delta _8^2-4 \delta _5 \delta _7}\right) \nonumber \\ &  \quad \quad f'\left( y+\frac{\left( \sqrt{\delta _8^2-4 \delta _5 \delta _7}-\delta _8\right) z}{2 \delta _7}\right) -2 \delta _7 h'(t)]/\delta _7]/(4 \delta _6 \phi ). \end{aligned}$$

**Figure 9:**
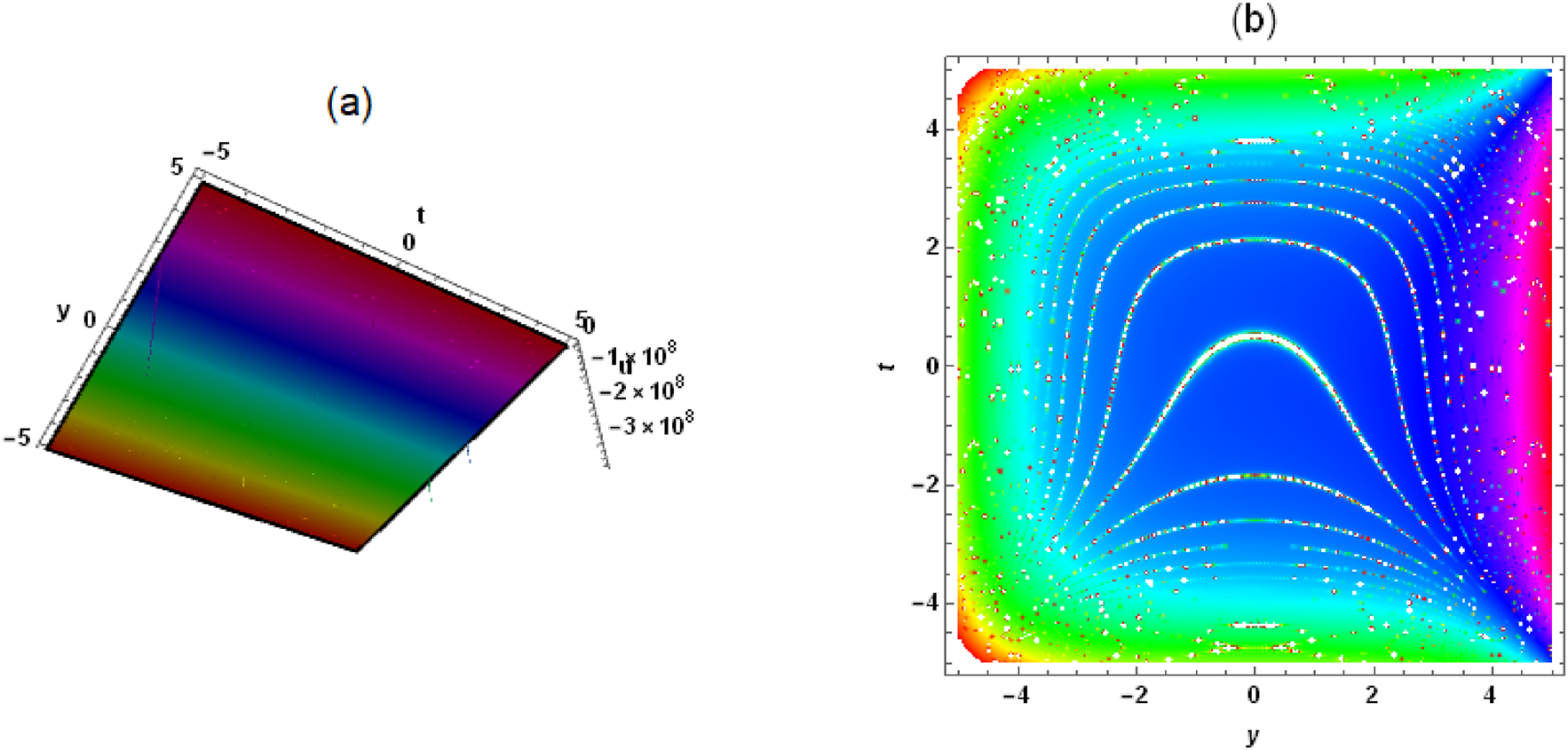
Solution (17) with $\delta _1=\delta _2 =\delta _3=\delta _4=\delta _5=1$, $C_2=-1$, $\delta _8=2$, $C_1=\delta _6=\delta _7=\lambda =\mu =1$, $\phi =1/4$, $z=x=0$, (**a**) three-dimensional plot, (**b**) density.

In solution (15)-(17), $$f(y+c z)$$ and *h*(*t*) are not restricted in any way and can be arbitrarily evaluated, which leads to an infinite number of exact solutions to Eq. (1), both linear and nonlinear, which is very interesting. To understand the physical structure of these solutions, let’s take solution (17) as an example. When $$f(y+c z)=y+\frac{\left( \sqrt{\delta _8^2-4 \delta _5 \delta _7}-\delta _8\right) z}{2 \delta _7}+1$$ and $$h(t)=3 t+2$$, a periodic wave structure can be seen in Fig. 7. It’s a traveling wave solution. When $$f(y+c z)=\left( y+\frac{\left( \sqrt{\delta _8^2-4 \delta _5 \delta _7}-\delta _8\right) z}{2 \delta _7}\right) ^2+1$$ and $$h(t)=t^2+2$$, solution (17) presents a very interesting structure (see Fig. 8). When $$f(y+c z)=\cosh \left( y+\frac{\left( \sqrt{\delta _8^2-4 \delta _5 \delta _7}-\delta _8\right) z}{2 \delta _7}\right) 
+1$$ and $$h(t)=\sinh (t)+2$$, a new physical structure can be seen in Fig. 9.

*Case 2:*

18 $$\begin{aligned} &  a_2=-\frac{6 \delta _4 \phi ^2}{\delta _6}, a_1=-\frac{6 \delta _4 \lambda \phi ^2}{\delta _6}, f(y+c z)=\chi _1 (c z+y)+\chi _2,\nonumber \\ &  \quad a_0=-[f'(c z+y) [\phi \left( c \delta _2+\delta _1\right) +[c \left( c \delta _7+\delta _8\right) +\delta _5] f'(c z+y)]\nonumber \\ &  \quad \quad +\,\delta _4 \phi ^4 \left( \lambda ^2+8 \mu \right) +\delta _3 \phi ^2+\phi h'(t)]/(2 \delta _6 \phi ^2), \end{aligned}$$

where $\chi _1$ and $\chi _2$ are integral constants. Substituting Eqs. (11)-(13) and Eq. (18) into Eq. (9), respectively, gives Eq. (1) another set of rational solution, hyperbolic function solution and periodic solutions. We won’t go into details here.

Due to the arbitrariness of the *f* and *h* functions in solutions (15)-(17), we can obtain an infinite number of exact solutions for traveling and non-traveling waves in Eq. (1), which was not possible with previous traveling wave transformations in the $(G'/G)$-expansion method, greatly promoting the development and application of this method.

## Conclusion

   In this work, a (3 + 1)-dimensional Painlevé integrable equation is investigated in fluid mediums, which was first proposed by Prof. Abdul-Majid Wazwaz. The Painlevé integrable equation has a profound background in physics. These equations have wide applications in many scientific fields, especially in physics. For example, in fields such as quantum mechanics, statistical physics, nonlinear optics, and celestial mechanics, many important models can be reduced to differential equations that satisfy the Painlevé property. The interaction solution between lump wave and solitary wave are obtained by utilizing the Hirota bilinear form and Mathematica software^36–43^, which has not been studied in other literature yet. Figures 1 and 2 show the interaction between lump wave and single soliton. From the beginning of their respective propagation to mutual fusion, the amplitude of lump wave decreases continuously, which is an inelastic collision. Figures 3 and 4 depict the interaction between a lump wave and two solitary waves, propagating from one soliton to the other with amplitudes from small to large and then from large to small. Meanwhile, the interaction solution between lump wave and periodic wave are also presented, which is shown in Figs. 5 and 6. This type of interaction solution is also being discussed for the first time. Finally, a rich set of exact solutions for the (3 + 1)-dimensional Painlevé integrable equation are derived by using the $(G'/G)$-expansion method, which includes rational, hyperbolic function and trigonometric function solutions. Because of the arbitrariness of $f(y+c z)$ and *h*(*t*), we can get infinitely many exact solutions to Eq. (1). Some new physical structures are found in Figs. 7, 8 and 9. The $(G'/G)$-expansion method has the characteristics of simple calculation and accurate solution. By constructing specific expansions, complex nonlinear equations can be transformed into simple algebraic systems, thereby reducing the difficulty of solving. In addition, this method can also obtain multiple solutions to nonlinear equations, including soliton solutions, periodic solutions, etc., providing rich mathematical tools for the study of nonlinear phenomena. We will replace the linear transformation in the $(G'/G)$-expansion method with a nonlinear transformation, which can obtain more exact solutions of different types but also increase the difficulty of solving. Moreover, the nonlinear transformation used is not universal, and different equations require different types of nonlinear transformations. In the future, we hope to find a unified nonlinear transformation to solve most nonlinear problems. All the results of the paper have been validated for accuracy through Mathematica software.

## Data Availability

Data sharing not applicable to this article as no datasets were generated or analysed during the current study.
